# French Anti-Tuberculosis Measures

**Published:** 1917-05-19

**Authors:** 


### FRENCH ANTI-TUBERCULOSIS MEASURES.

A RECENT number of the old-established *Journal de Médecine de Bordeaux* is "consecrated" (the word may, schoolboy fashion, be given its English double in such a connection) to the anti-tuberculosis campaign in the region of the Gironde, particularly as regards tuberculous soldiers. What first takes one's attention is the variety of institutions at disposal. If in this country we have four or five "links" in the chain of agencies, the sanatorium, dispensary, hospital, farm colony, open-air school, let us say, then in the south-east of France there seem to be nearly a dozen. There is the "Station forestière et maritime d'Arcachon," the sanatorium, for adults and for children, for pulmonary and for surgical tubercle, the marine and heliotherapeutic station, the tuberculosis dispensary founded in 1903—one is apt to forget that on the Continent France is regarded as the home of the dispensary movement—the "œuvre de la préservation de l'enfance contre la tuberculose," and lastly the model detached cottage.

### SUNSHINE AND FRESH AIR.

Of these "links" the first named was well known long before the war. The reclaimed foreshore in the neighbourhood of Arcachon became, perhaps unexpectedly to its creators, a health resort. Sandy and pine-grown, it used to be a favourite winter station for English consumptives of means. They practised the "cure libre," such as obtains often still, indeed too much, in Switzerland or Bournemouth; there was no sanatorium restraint, but reclining-chairs stood outside many houses and hotels, the local medical men paid visits, and Arab saddle ponies could be hired cheaply to convey the invalid over the shifting soil. The chief sanatorium is the large institution at Pessac, with 135 beds. It was founded in 1899, and at the outbreak of war a good number of old patients, discharged from the sanatorium ten years before, applied for a certificate to that effect when called up. Others fought, indeed in some instances for long periods in the trenches, and a few obtained the Croix de Guerre. The institutions for surgical tuberculosis are founded on the model of the well-known one at Berck-sur-Mer, whose methods were also copied at our own Cripples' Home at Alton in Hampshire. The district of the Gironde must be very favourable to heliotherapy, and high percentages of cure are claimed for every common kind of surgical tuberculosis except Pott's disease. The "œuvre de la préservation de l'enfance contre la tuberculose" is a purely prophylactic affair. The medical staff visit tuberculous families, pick out the sound children, and send them away into the country to be boarded out with private families. The principle of this procedure is that adopted and advocated years ago by Bang of Copenhagen, to secure the rearing of a tubercle-free herd of milch cattle, and recently practised by an English municipality. In 1906 the yearly cost of maintenance of a child was £16. The model dwelling movement was set on foot in 1893, in order to provide working-class families with cheap, healthy houses, detached and provided with gardens. Among 130 families thus accommodated in ten years, there have been, it is stated, only eleven cases of tuberculosis, and no family has lost more than one child from tuberculosis. In consequence of, or more correctly, in succession to, these various efforts against the disease, it has, as everywhere else, declined steadily at least as regards the pulmonary form.

### MOBILISATION AGAINST TUBERCULOSIS.

Not that there failed a careful war mobilisation of anti-tuberculosis work. There does not seem to have been, as in Germany, a special tuberculosis section of the Red Cross. The procedure was to send the tuberculous soldier, or the soldier suspected of tuberculosis, to an approved sanatorium, or to one of the five approved "sanitary hospitals," for treatment or diagnosis. There he remained as long as the disease was acute. When somewhat recovered, the invalid leaves for the "sanitary station," again at a private institution, where for three months he has anti-tuberculosis treatment and education. After that period he comes before what we should term a medical board, which either sends him back to service, temporarily or indefinitely, or to auxiliary service, or discharges him from the Army. In the last event, the prefect of the department in which he takes up his residence is notified, and the patient is looked after by a new body inaugurated by the Minister of the Interior—namely, the departmental committee of help for military tuberculous patients, which in some instances has a sub-committee in every arrondissement. Money or food grants may be given, spitting-flasks or other surgical aid furnished, and children exposed to infection boarded away from home. On the whole, however, the funds available for these purposes are not sufficient in practice. A military tuberculosis home for the dying was not in the case of the Gironde deemed advisable. The difficulties of prognosis are notoriously great in consumption, so that it is impossible to pick out a sufficient number of patients with only a short time to live; moreover, the depressing atmosphere of such an institution was deemed a great objection. Nor is any proposal recorded for erecting national military sanatoriums, such as existed in America before the war, and were talked of in this country when Colonel Seely was War Minister. Probably the great numbers of consumptive soldiers now presenting would make such a plan impracticable. However, one learns that the "sanitary stations" have not enough accommodation. Human nature is uniform, and the same unwillingness of tuberculous soldiers to stop long enough at the sanatorium has been noticed in France as in England. The chief new organisation desiderated is one which should secure to cases of arrested disease a light rural occupation. The suggested "bureau de placement à la campagne pour tuberculeux réformés" is indeed for France, with her initially scanty, now further hugely depleted, population, almost a necessity. What is adumbrated is the small-holding system—which, one would think, should suit national usages very well. The traditional English countryside may be visibly changed from the same cause, "shoots" in Norfolk, and fox preserves in the Midlands, yielding to liberal-sized allotments and to co-operative colonies, rearing rosy-faced children, but visited, alas! by the motor-'bus.

